# Diapause and Anoxia-Induced Quiescence Are Unique States in Embryos of the Annual Killifish, *Austrofundulus limnaeus*

**DOI:** 10.3390/biom15040515

**Published:** 2025-04-01

**Authors:** Patrick R. Clouser, Claire L. Riggs, Amie L. T. Romney, Jason E. Podrabsky

**Affiliations:** 1 Department of Biology, Center for Life in Extreme Environments, Portland State University, P.O. Box 751, Portland, OR 97201, USA; arom2@pdx.edu; 2 Division of Rheumatology, Inflammation, and Immunity, Brigham and Women’s Hospital, Boston, MA 02115, USA; clriggs@bwh.harvard.edu; 3 Department of Medicine, Harvard Medical School, Boston, MA 02115, USA

**Keywords:** dormancy, stress, transcriptomics, development, killifish

## Abstract

Diapause is a state of developmental and metabolic dormancy that precedes exposure to environmental stresses. Yet, diapausing embryos are typically stress-tolerant. Evidence suggests that diapausing embryos “prepare” for stress as part of a gene expression program as they enter dormancy. Here, we investigate if diapause II embryos of the annual killifish *Austrofundulus limnaeus*, which can survive for hundreds of days of anoxia, can mount a transcriptomic response to anoxic insult. Bulk RNAseq was used to characterize the transcriptomes of diapause II embryos exposed to normoxia, 4 h and 24 h anoxia, and 2 h and 24 h normoxic recovery from anoxia. Differential expression and gene ontology analyses were used to probe for pathways that may mitigate survival. Transcriptional factor analysis was used to predict potential mediators of this response. Diapausing embryos exhibited a robust transcriptomic response to anoxia and recovery that returns to near baseline conditions after 24 h. Anoxia induced an upregulation of genes involved in the integrated stress response, lipid metabolism, p38mapk kinase signaling, and apoptosis. Developmental and mitochondrial genes decreased. We conclude that diapause II embryos mount a robust transcriptomic stress response when faced with anoxic insult. This response is consistent with mediating expected challenges to cellular homeostasis in anoxia.

## 1. Introduction

Metabolic dormancy is deployed across the tree of life to synchronize activity, reproduction, and development with favorable environmental conditions [1,2,3]. Diapause is a state of developmental and metabolic dormancy that occurs during embryonic development in a variety of animal species [3]. Diapause is considered an endogenously promoted dormancy, which means that internal signals—typically considered part of the developmental program—control entrance into dormancy. Thus, diapause dormancy occurs under conditions conducive to normal development, although environmental signals and factors are typically involved in the induction of diapause. Stress tolerance, sometimes even extreme stress tolerance, almost always co-occurs with diapause [4]. Due to the general physiological characteristics of most diapausing embryos, such as severely reduced rates of ATP turnover and protein synthesis, it is somewhat paradoxical that they exhibit increased tolerance to environmental stresses that require a robust and complex physiological and molecular response in active animals. Thus, it is assumed that diapausing embryos “prepare” for stress as part of a gene expression program as they enter dormancy. Indeed, there is a great deal of evidence supporting the accumulation of molecular and chemical chaperones and the alteration of metabolic pathways during the entry phase of diapause [5,6,7,8,9,10]. However, few studies have profiled global gene expression responses to stress in diapausing embryos. In this study, we challenge diapausing annual killifish embryos with anoxic stress to test the hypothesis that they are already poised to tolerate stress and will lack a typical transcriptional response.

While there are many species of annual killifishes that produce diapausing embryos, the most well-studied species in terms of the physiological, biochemical, and molecular aspects of diapause and stress tolerance is *Austrofundulus limnaeus* [11,12]. Populations of *A. limnaeus* survive in ephemeral ponds by producing embryos that can enter embryonic diapause at three distinct developmental stages, termed diapause I, II, and III [13,14]. Entry into diapause II, which occurs midway through development, is a unique developmental trajectory with patterns of gene expression that diverge from actively developing embryos many days prior to the cessation of development [15]. During this period, embryos experience an increase in tolerance to environmental stresses such as oxygen limitation and osmotic stress [11,16,17,18,19]. The gene expression program associated with entrance into diapause II is presumed to prepare the embryos for survival of the harsh conditions associated with the dry season in their native habitat, because once in diapause, embryos have a severely reduced metabolic rate and experience over a 90% reduction in the rate of protein synthesis [20,21].

Embryos of *A. limnaeus* are the most anoxia-tolerant vertebrates [11,19,22,23,24]. Diapause II embryos can survive for hundreds of days without oxygen and have an LT_50_ of 62 days of anoxia at 25 °C [25]. Embryos of *A. limnaeus* respond quickly to removal of oxygen. When diapause II embryos are exposed to anoxia, heat dissipation decreases slightly but not significantly over the first 16 h, suggesting that metabolism remains relatively constant across the transition into anoxia [26]. However, ATP levels fall by 50%, and various indices of energetic status suggest a severely energy-limited system after only 14 h of anoxia [26]. Amazingly, embryos continue to survive for months with severely reduced ATP levels. Thus, we hypothesized that diapausing embryos of *A. limnaeus* will be prepared for stress and have a muted or non-existent stress response during initial exposure to anoxia. Alternatively, if they do respond to anoxic stress, it will most likely occur within the first 24 h of exposure and before the large-scale reductions in ATP are complete. Here we report that diapausing embryos respond with a strong transcriptomic response within 24 h of anoxia. This paper highlights the players that likely orchestrate the early responses to oxygen limitation in this species and may explain the extreme stress tolerance in these embryos.

## 2. Materials and Methods

### 2.1. Embryo Rearing and Staging

Embryos were incubated at 25 °C in the dark, and embryo medium was changed daily. At 4 days post-fertilization (dpf), the embryos were treated with two 5 min washes in 0.03% sodium hypochlorite (Clorox bleach, Clorox company, Oakland, CA, USA), as previously described [27]. Following bleaching, the embryos were incubated in embryo medium containing 10 μg/L gentamicin sulfate. Embryos were staged according to Wourms’ staging regime; embryos typically enter diapause II at 24 days post-fertilization (dpf) when incubated at 25 °C [14,28].

### 2.2. Anoxic Exposure and Aerobic Recovery

**Figure 1:**
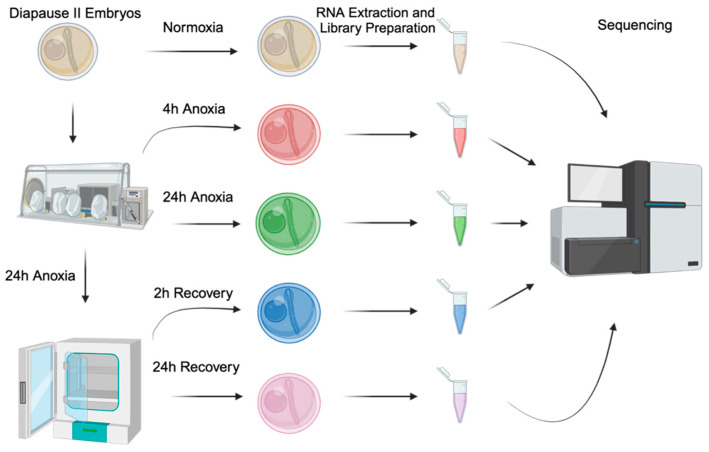
Experimental design and workflow. Experimental conditions include the following: normoxia (t = 0, tan), 4 h of anoxia (red), 24 h of anoxia (green), 2 h of normoxic recovery (green), and 24 h of normoxic recovery (pink). All samples are a pool of 20 embryos, and 4 different spawning events were sampled (n = 4).

Diapause II embryos at 32 dpf were exposed to 24 h of anoxia at 25 °C in the dark in a Bactron III anaerobic chamber (Sheldon Manufacturing, Cornelius, OR, USA), as previously described [25,29]. This methodology immediately exposes embryos to an anoxic environment. Aerobic recovery was initiated by removing the embryos from the chamber and rinsing them three times in normoxic embryo medium. Recovering embryos were incubated at 25 °C in the dark until sampling [25]. Embryos were sampled at the following time points: normoxia (t = 0), 4 h and 24 h anoxia, and after 2 h and 24 h of aerobic recovery (Figure 1). Experimental trials were repeated on 4 separate spawning events (n = 4). Each time point/sample consisted of groups of 20 pooled embryos. For qPCR validation of RNAseq results, only the normoxic and 24 h anoxia time points were replicated in diapause II embryos that were 55 dpf (n = 3–4).

### 2.3. Sampling Embryos for RNA Sequencing and qPCR

To sample, embryos were blotted dry on a 100 µm nylon mesh screen, and excess embryo medium was wicked away on a paper towel. Groups of 20 embryos were transferred to a pre-weighed microcentrifuge tube and flash-frozen in liquid nitrogen. This procedure was performed within the anaerobic chamber for anoxic samples that were immediately flash-frozen upon removal from the anaerobic environment [25]. Frozen samples were stored at −80 °C until RNA extraction.

### 2.4. RNA Extraction

Total RNA was extracted from each sample with TRIzol reagent (Invitrogen, Carlsbad, CA, USA), as previously described [25,30,31,32,33]. RNA was precipitated by overnight incubation in a high-salt solution at −20 °C according to the manufacturer’s instructions [32], and samples were resuspended in 1 mM sodium citrate (pH = 6.5). Concentration and purity of RNA were determined by UV spectroscopy using an a NanoQuant plate and an Infinite Pro M200 plate reader (Tecan, San Jose, CA, USA; Table S1). RNA integrity was assessed using agarose gel electrophoresis. RNA samples were stored at −80 °C until library preparation. The RNA samples used in this study are the same as those used to previously profile small noncoding RNAs in response to anoxia [25].

### 2.5. Poly-A RNA Sequencing

cDNA libraries for sequencing were prepared using the Illumina TruSeq RNA Sample Preparation Kit (v2, Illumina, San Diego, CA, USA) according to the manufacturer’s instructions with a starting input of 1 μg of total RNA. The quality of the purified cDNA libraries was assessed using an Agilent 2100 Bioanalyzer equipped with a DNA 1000 chip (Agilent Technologies, Santa Clara, CA, USA). Quantitative PCR was used to measure cDNA content. Libraries were sequenced (100 nt paired-end reads) on an Illumina HiSeq 2000 at the Oregon Health & Science University’s Massively Parallel Sequencing Shared Resource Facility.

### 2.6. Analysis of Sequence Data

Sequence data were analyzed as previously reported for transcriptomic analysis in *A. limnaeus* [34]. The bioinformatic pipeline was performed on a Linux high-performance computing resource at PSU. Read data quality was assessed using FastQC (version 0.10.1) to ensure high-quality sequencing results for each sample [35]. Poor-quality sequence reads and adaptors were removed with Trimmomatic (v 0.33) using the following settings: “ILLUMINACLIP:2:30:7:1:true”, “SLIDINGWINDOW:5:15”, “LEADING:20”, “TRAILING:20”, and “MINLEN: 25” [36]. Resultant reads were mapped to the *A. limnaeus* genome with Bowtie2 (v 2.5.1) using preset parameters for very fast local alignments [37]. Only reads that aligned with 0 mismatches were used. Gene counts were generated with the R/Bioconductor software package GenomicAlignments (ver 1.42.0) in union mode with the *summarizeOverlaps* function [38] in conjunction with the NCBI *A. limnaeus* genome annotation Release 100 [39].

### 2.7. Differential Gene Expression Analysis

Differential expression analysis was performed using the R package DEseq2 (v 1.46.0) [40]. Gene count data were initially prefiltered to remove genes with row sum totals across all conditions with less than 5 counts and used to create a DEseq data set object. Principal component analysis was also performed with DEseq2 to assess the variance between all experimental conditions. Differentially expression data were acquired at the gene level for all experimental conditions by pairwise comparison to normoxic (t = 0) samples. Transcripts with a *p*-value less than 0.05 (Benjamini–Hochberg corrected for multiple comparisons) and a log_2_ fold change ± 0.58 were considered differentially expressed [40].

### 2.8. Quantitative Real-Time Polymerase Chain Reaction (qPCR)

The original RNA samples used to generate the RNAseq data were lost due to a freezer failure, so new RNA samples were generated as described above using diapause II embryos exposed to normoxia and 24 h of anoxia. Total RNA in the samples was quantified using a NanoDrop One spectrophotometer (ThermoFisher, Scientific, Waltham, MA, USA). Single-stranded cDNA libraries were generated using a Qiagen reverse transcriptase kit (RT-020, Qiagen, Hilden, Germany) using 85 ng of total RNA as the starting material in a 20 μL reaction. Samples were incubated for 10 min at 25 °C, 30 min at 50 °C, and 5 min at 85 °C in a thermocycler (Geneamp 2700 thermocycler, Applied Biosystems, Waltham, MA, USA) and then immediately placed on ice. qPCR (Applied Biosystems Vii7 Real-Time PCR system, Applied Biosystems, Waltham, MA, USA) was performed on 10 μL reactions in a 386-well plate format. Each reaction consisted of 5 μL of Luna universal PCR master mix (New England biolabs, Ipswich, MA, USA), 0.25 μL each of forward and reverse primers (Integrated DNA Technologies, Coralville, IA, USA), 3.5 μL nuclease-free water, and 1 μL of ss cDNA template. Reactions were subjected to an initial denaturation step at 95 °C for 60 s, followed by 40 cycles of denaturation for 15 s at 95 °C and extension for 30 s at 60 °C. Melting curves were performed after each reaction to confirm a single PCR product. At least 2 technical replicates were performed for each sample, on 3–4 biological replicates. Relative fold-change values between normoxia and 24 h anoxia were calculated using the ΔCt method with the mean Ct values for normoxic samples serving as the reference for each anoxic sample.

### 2.9. Ortholog Mapping and Gene Ontology Analysis

Orthologous proteins were identified between *A. limnaeus* and *Homo sapiens* using NCBI BLASTp and the R package OrthoFinder (v 2.5.5) [41]. Sequences for all *A. limnaeus* proteins were aligned to the GHR38 human protein database retrieved from ENSMBL, keeping the top returned result with an e-value less than 0.0001. The same *A. limnaeus* protein sequences were also analyzed against the human genome with OrthoFinder [41]. The two returned lists were then merged, and any duplicate *A. limnaeus* proteins found associated to the same transcript were removed.

Gene ontologies analyses were generated using the R library clusterProfiler (v 4.14.4) [42], with the *H. sapiens* database from annotationDBi (v 1.68.0). Differential expression data from DEseq2 for each of the experimental conditions were matched to the ENSMBL ID of the *H. sapiens* ortholog list described above. Overrepresentation analysis (ORA) was used to return the associated statistically significant gene ontology terms for biological process, molecular function, and cellular component (*p*-values < 0.05). The simplify function in clusterProfiler was used to remove redundant terms (cutoff = 0.5, measure = “Wang”). In addition, KEGG pathways that were enriched in the differentially expressed genes were also identified within clusterProfiler. Relationships between the sets of differentially expressed genes in each treatment (and their associated gene ontologies and KEGG pathways) were compared using clusterProfiler’s compareCluster function.

### 2.10. Transcription Factor Analysis Using TFLink

Differentially expressed genes were interrogated for patterns that may implicate the action of known transcription factors (TFs) using the TFLink website [43]. Transcription factors that had significant associations with any of the up- or downregulated genes (with human orthologs) were identified for each experimental time point (*p* adjust value < 0.05).

### 2.11. Data Visualization

All graphs and tables were created using R packages ggplot2 (v 3.5.1) and clusterProfiler (v 4.14.4), GraphPad Prism (v 10.4.1), and Biorender (www.biorender.com).

## 3. Results

### 3.1. Sequencing Library Quality and Coverage

Illumina sequencing yielded an average of 49.1 million paired reads per cDNA library. Of these reads, an average of 93% were mapped to the *A. limnaeus* genome with zero mismatches after trimming and filtering. This depth of sequencing represents an average of 35X coverage of the genome in the expressed transcriptome. A total of 26,401 unique gene sequences were identified in the data set. A full table of sequencing results for each cDNA library can be found in Table S1.

### 3.2. Characterization of the Normoxic Diapause II Transcriptome Profile

**Figure 2:**
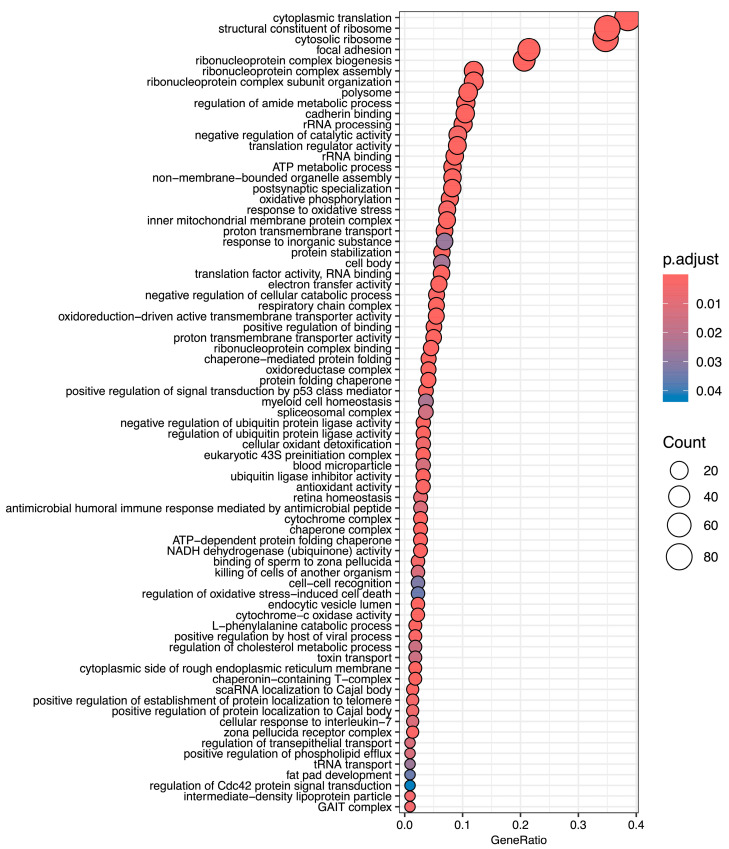
Gene ontology terms enriched in highly expressed genes in diapause II embryos of *Austrofundulus limnaeus*. Notable biological process terms are associated with protein expression, mitochondrial metabolic functions, and negative regulation of catabolism. Top molecular function terms of note include ribosomal and translational regulation, electron transport, ubiquitin ligase inhibitor activity, and protein folding. Terms listed here are limited to the highest possible term in the hierarchy.

Normoxic diapause II embryos (t = 0) had an average FPKM of 49 ± 394 (mean ± S.D.) reads per transcript. Transcripts equal to and above one-half standard deviation of the mean (245 reads) were denoted as “highly expressed” in normoxic diapause II embryos. This gene list consists of 271 transcripts, 245 of which map to *Homo sapiens* orthologs (Table S2). An enrichment analysis of all gene ontology terms (biological processes, molecular functions, and cellular component) based on human annotations that were enriched in this gene set are listed in Figure 2. Enriched cellular components of note include cytoplasmic ribosomes, polysomes, focal adhesions, several mitochondrial components, the spliceosome complex, and the GAIT complex (Figure 2). Of note, the third most abundant transcript expressed in diapause II embryos is a long non-coding RNA (LOC106513764) with unknown function.

### 3.3. Overall Characterization of Experimental Treatments

**Figure 3:**
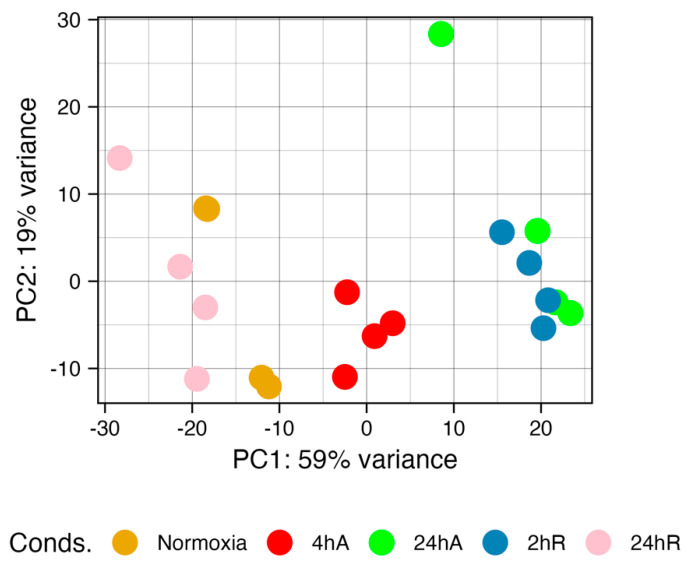
Principal component analysis of gene expression data for diapause II embryos of *Austrofundulus limnaeus* exposed to anoxia and recovery from anoxia. Gene expression patterns are similar in the 24 h of anoxia (24 hA) and 2 h (2 hR) embryos. Normoxic (t = 0) and 24 h of aerobic recovery (24 hR) embryos also share similar gene expression patterns. The 4 h of anoxia samples (4 hA) are the most unique. The proportion of the variance captured by each principal component suggests that the first two principal components explain around 80% of the variation in the data.

Principal component analysis reveals a separation of gene expression patterns as embryos respond to and recover from anoxia (Figure 3). Normoxic (t = 0) embryos and embryos after 24 h of aerobic recovery group together, suggesting a return to “control” gene expression patterns within 24 h of recovery. Gene expression patterns after 24 h of anoxia and at 2 h of aerobic recovery group also group together. The most distinct group of samples are those at 4 h of anoxia.

### 3.4. Differential Gene Expression

**Figure 4:**
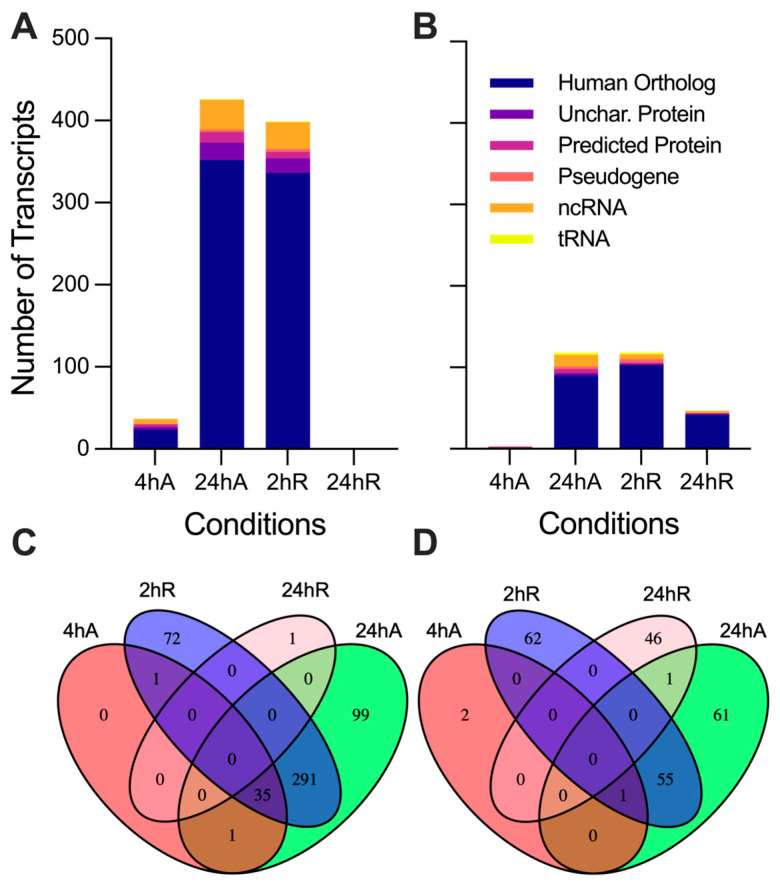
Differential gene expression patterns for diapause II embryos of *Austrofundulus limnaeus* exposed to anoxia and recovery from anoxia. Transcripts that increase in abundance (**A**) during anoxia and recovery from anoxia outnumber transcripts that decrease in abundance (**B**). Venn diagrams indicate the number of transcripts shared across experimental treatment groups that are (**C**) upregulated and (**D**) downregulated across the 24 h or exposure to and recovery from anoxia. Differential expression was determined by an adjusted *p*-value < 0.05 and a log_2_ fold change of ±0.58 (1.5-fold change).

**Figure 5:**
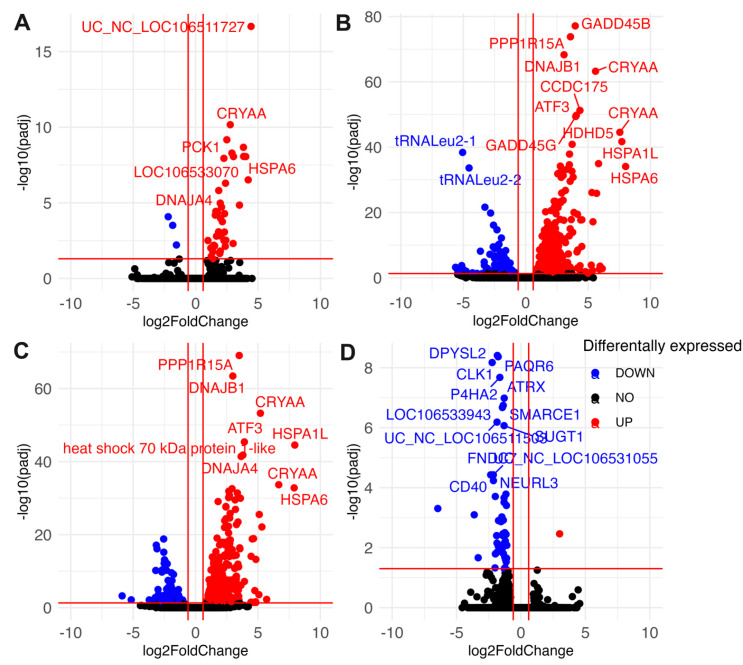
Volcano plots indicating differentially abundant transcripts in diapause II embryos of *Austrofundulus limnaeus* exposed to anoxia and recovery from anoxia. Transcripts differentially expressed after (**A**) 4 h of anoxia, (**B**) 24 h of anoxia, (**C**) 2 h of aerobic recovery from 24 h of anoxia, and (**D**) 24 h of aerobic recovery from 24 h of anoxia. Data points in blue significantly decrease in abundance, while those in red increase in abundance. Black data points are not statistically significant. Differential expression was determined by an adjusted *p*-value < 0.05 and a log_2_ fold change of ±0.58 (1.5-fold change).

Differentially expressed transcripts were split into five categories: those with identified human orthologs, uncharacterized proteins in NCBI, NCBI predicted proteins, tRNAs, and noncoding RNAs as described by the NCBI’s annotation of the *A. limnaeus genome* (Figure 4). A full list of differentially expressed transcripts is provided in Table S3 (up-regulated) and Table S4 (down-regulated). Differential gene expression patterns determined by DEseq2 and qPCR were consistent across platforms for a handful of genes validated by qPCR (Table S5). The greatest number of differentially expressed transcripts, relative to 0 h anoxia, were observed after 24 h of anoxia, with a total of 544 genes differentially expressed (up and down) (Figure 4A,B). Interestingly, only 37 transcripts were in higher abundance after 4 h of anoxia, and the majority of those were shared with upregulated transcripts after 24 h of anoxia and 2 h of recovery (Figure 4 and Figure 5). This relatively small number of immediately up-regulated transcripts contains genes with some interesting functions, such as phosphoenolpyruvate carboxykinase, lipase E, iodothyronine deiodinase 2, molecular chaperones and heat shock proteins, and several genes of unknown function (Figure 5A). Only one transcript from an uncharacterized locus in *A. limnaeus* was uniquely upregulated after 4 and 24 h of anoxia, LOC106533231. This locus codes for a gene with significant similarity to human *kmt5c*, a histone methyltransferase. Over 75% of the transcripts that increased in abundance are shared between 24 h of anoxia and 2 h of aerobic recovery, and about 47% of transcripts that decreased in abundance are shared across these two time points. Interestingly, only one transcript was still upregulated after 24 h of recovery from anoxia, a locus with unknown function named protein TsetseEP-like (Figure 5D).

Somewhat surprisingly, far fewer transcripts decreased in abundance in response to anoxia compared to those that increased (Figure 4B and Figure 5A). Notably, only three transcripts immediately decreased in abundance in response to anoxia, transcripts for regulator of G-protein signaling-2 (*rgs-2*), taxilin γ (*txlng*), and a pseudo 16S transcript from the mitochondrial genome (Figure 5A). Downregulated RNAs also contained a higher proportion of noncoding RNAs and tRNAs compared to upregulated RNA species, with three mitochondrially encoded transcripts, *tRNALeu2-1*, *tRNALeu2-2*, and a segment of the pseudo16S RNA, being strongly decreased (Figure 5A–C). Interestingly, transcripts that are suppressed by anoxia do not appear to return to normoxic values, with almost 40% of the genes still in lower abundance compared to controls after 24 h of recovery (Figure 4B and Figure 5D). Further, downregulated transcripts appear to have a more time-point specific expression pattern compared to those that are upregulated, as evidenced by the 46 transcripts uniquely downregulated only at 24 h of recovery (Figure 4D and Figure 5D).

### 3.5. Gene Ontology Overrepresentation Analysis of Differentially Abundant Transcripts

**Figure 6:**
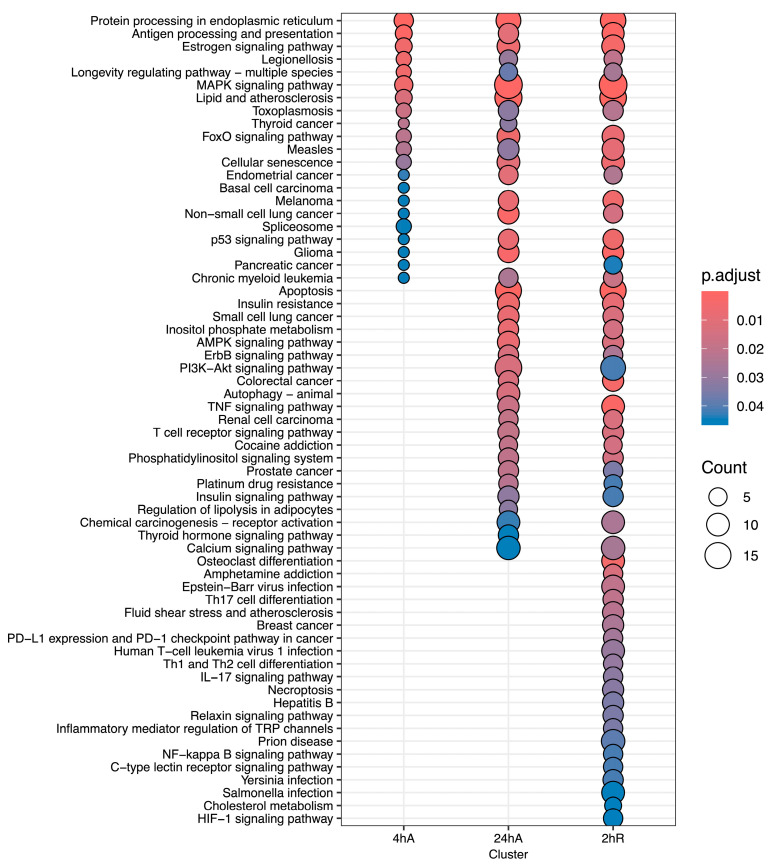
KEGG pathway analysis for genes that increase in abundance in response to anoxia and recovery from anoxia in diapause II embryos of *Austrofundulus limnaeus*.

**Figure 7:**
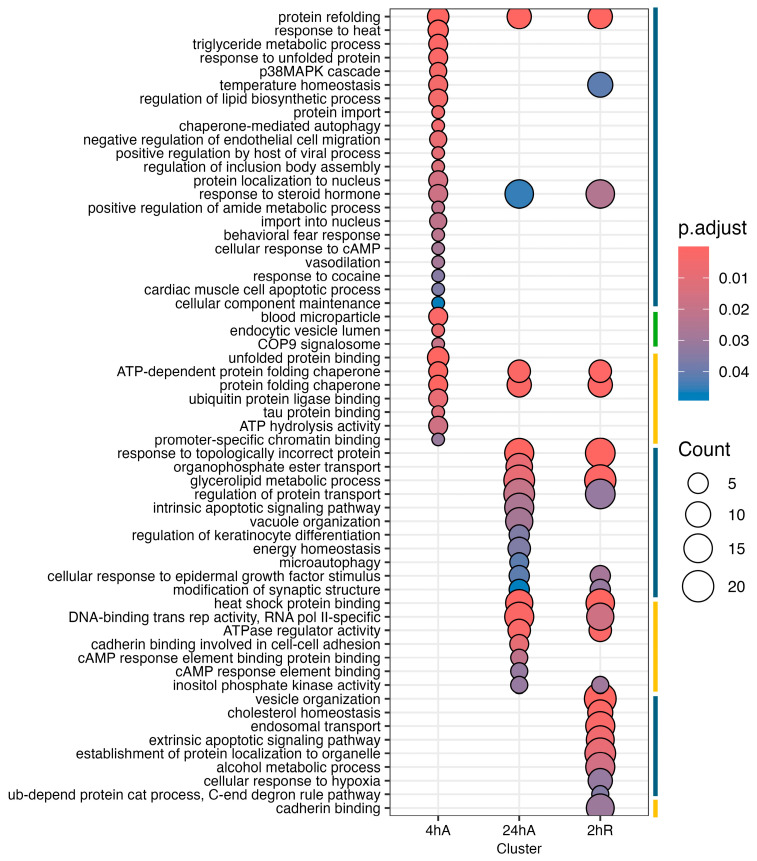
Gene ontology enrichment analysis for genes that increase in abundance in response to anoxia and recovery from anoxia in diapause II embryos in *Austrofundulus limnaeus*. The colored bar on the right side of the graph represents ontology family: blue = biological process, yellow = molecular function, and green = cellular component.

**Figure 8:**
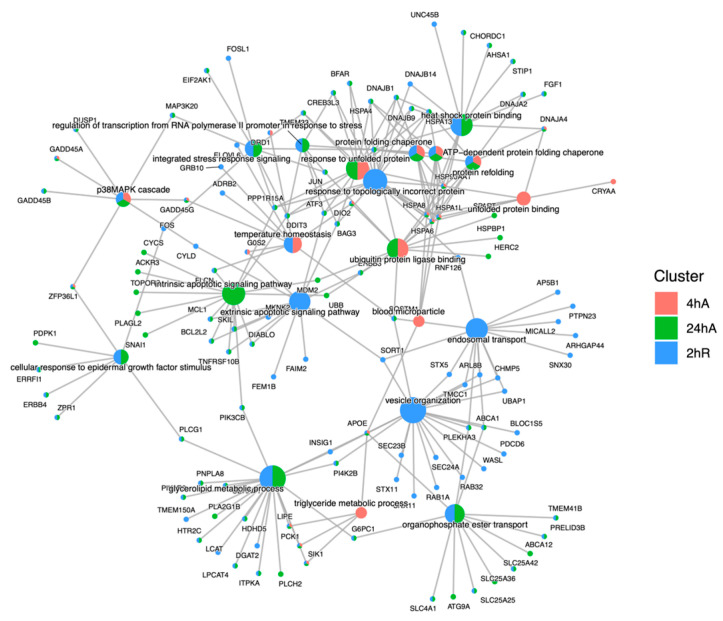
Cnet plot of biological process gene ontology enrichment analysis for genes that increase in abundance in response to anoxia and recovery from anoxia in diapause II embryos of *Austrofundulus limnaeus*. Central nodes represent ontology terms, while peripheral nodes are genes associated with the ontology term.

Ortholog mapping resulted in 85% of the *A. limnaeus* proteins being mapped to human orthologs. For the differentially expressed transcripts, over 83% of transcripts were mapped to human orthologs, with an additional 9% mapping to non-coding RNAs described in the *A. limnaeus* genome. Figure 6 presents dot plots of KEGG pathways, and Figure 7 shows gene ontology terms overrepresented in lists of transcripts that increased in abundance in experimental compared to control conditions (t = 0). These pathways are highly interrelated and appear to build over time, as illustrated in Figure 8. A full list of upregulated transcripts that contribute to the KEGG and gene ontology terms are provided in Tables S6 and S7, respectively. KEGG pathway overrepresentation appears to increase over the time course of exposure to anoxia and during early recovery, suggesting a sequence of immediately acting and later-acting pathways that build on each other. The most significantly overrepresented KEGG pathways that initially respond to anoxia (4 hA) are associated with protein processing in the ER, estrogen signaling, the FoxO pathway, and a variety of cancers (Figure 6). By 24 h of anoxia, additional pathways associated with AMPK signaling, TNF-signaling, longevity, MAPK signaling, insulin signaling, and thyroid hormone signaling are overrepresented as well. Finally, during the initial recovery from anoxia, pathways associated with interleukin-17 signaling, necroptosis, NF-kappaB signaling, and HIF-1 signaling area additionally overrepresented (Figure 6). Gene ontology overrepresentation provides a slightly different pattern of functional analysis, with biological process terms being more uniquely overrepresented in different treatment groups (Figure 7). Not surprisingly, protein folding is strongly overrepresented in all groups, as is response to steroid hormones. Cell signaling pathways that are overrepresented during initial exposure to anoxia are p38MAPK cascade and cellular response to cAMP. Only during early recovery does cellular response to hypoxia become overrepresented.

**Figure 9:**
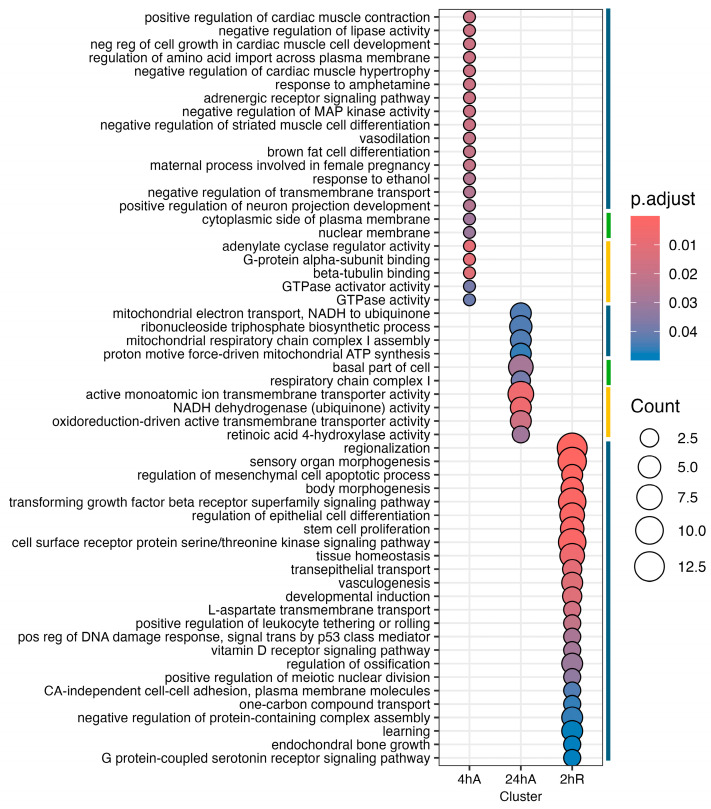
Gene ontology enrichment analysis for genes that decrease in abundance in response to anoxia and recovery from anoxia in diapause II embryos of *Austrofundulus limnaeus*. The colored bar on the right side of the graph represents ontology family: blue = biological process, yellow = molecular function, and green = cellular component.

**Figure 10:**
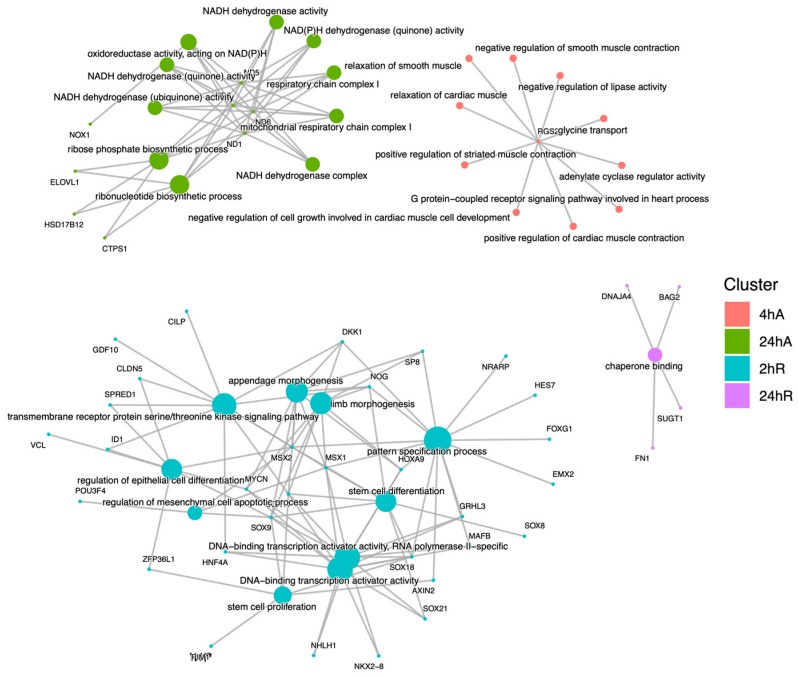
Cnet plot of biological process gene ontology enrichment analysis for genes that decrease in abundance in response to anoxia and recovery from anoxia in diapause II embryos of *Austrofundulus limnaeus*. Central nodes represent ontology terms, while peripheral nodes are genes associated with the ontology term.

Far fewer transcripts decreased in abundance in response to anoxia and recovery from anoxia, resulting in a less diverse set of overrepresented pathways and gene ontology terms (Figure 9, Table S8). Interestingly, genes that decreased in abundance do not appear to be interrelated but instead are specific to a single time point (Figure 10). After 24 h of anoxia, KEGG pathways associated with mitochondrial electron transport and ATP synthesis are overrepresented (Table S9). During early recovery from anoxia, KEGG pathways associated with development, stem cell proliferation, body morphogenesis, vasculogenesis, and vitamin D signaling are all overrepresented in the downregulated gene set. Gene ontology terms associated with biological process suggest a downregulation of G-protein coupled receptor activity and reduced electron transport processes in the mitochondrial membrane, as well as retinoic acid signaling and metabolism.

### 3.6. Transcription Factor Prediction

The set of differentially expressed genes initially responding to anoxia (4 h anoxia time point) are enriched for potential targets of 13 transcription factors (Table 1, adjusted *p* values < 0.05). The TF with the highest significance is peroxisome proliferator-activated receptor α (PPARA) which is associated with the regulation of seven of the upregulated transcripts at 4 h of anoxia (*lipE*, *gadd45g*, *gadd45a*, *g0s2*, *pck1*, *G0S2*, *CBFA2T3*). CBFA2T3, a target of PPARA, is a transcriptional coregulator associated with the modulation of RGS2, TRIM29, TXLNG, PCK1, DNAJA4, ZFP36L1, PAQR5, GADD45G, and HSPA8. Thus, PPARA and CBFA2T3 can account for a large portion of the differentially expressed genes in the initial response to anoxia. The TFlink database predicts 705 significant target interactions based on the gene sets associated with 24 h of anoxia; among the top returns are *TFAP4*, *TBP*, *KLF5*, *SMAD1*, and *E2F1* (Table 1). Gene expression patterns associated with the initial recovery from anoxia are associated with 752 significant transcription factors, with the most statistically significant being *SIN3A*. A full list of predicted transcription factors can be found in Table S10.

## 4. Discussion

The transcriptome of normoxic diapause II embryos is enriched for multiple gene ontologies associated with stress tolerance and protein synthesis, suggesting that preparation for stress may be part of the developmental program associated with entrance into diapause. While additional studies, including comparisons to actively developing embryos, are needed to fully evaluate the unique aspects of the diapause II transcriptome, there are some general trends that are worth highlighting. First, the transcript with the highest abundance in normoxic diapause II embryos is *HSPA8*. This finding agrees with previous research showing elevated levels of HSP70 proteins in diapause II embryos of *A. limnaeus* [44]. *HSPA8* is a constitutively expressed heat shock 70 transcript, which has a variety of functions in addition to its ability to act as a molecular chaperone [45], including a role in the normal development of anterior structures in zebrafish [46]. The expression of *HSPA8* prior to insult may be an indication of diapausing embryos’ preparedness to encounter and deal with environmental stress while in diapause, or it may indicate stabilization of a transcript that is needed to support immediate post-diapause II development. The latter conclusion is also supported by the domination of transcripts that encode for major components and regulators of cytoplasmic protein synthesis, RNA splicing, and oxidative phosphorylation. The high abundance of transcripts encoding ribosomal proteins and various translation initiation and elongation factors indicate that diapause II embryos may be prepared to synthesize and assemble the translation machinery in response to termination of diapause or to external stresses. Three mitochondrial transcripts, *co1*, *co3*, and *atp6*, are among the top 12 expressed genes in the diapause II transcriptome, which suggests active or at least stabilized mitochondrial physiology in dormant embryos. Thus, some aspects of the highly expressed transcriptome in diapause II embryos suggest preparation for stress, but perhaps the majority of transcripts appear to poise embryos for responses to internal or external signals with a robust gene expression response.

It is important to note that these experiments were conducted in a controlled laboratory environment that cannot mimic the natural conditions experienced by annual killifish embryos. In the wild, embryos would likely be exposed to multiple simultaneous stressors, and what appears as an inducible response in the lab may be a constitutive response in the field. However, this study was focused on creating controlled conditions that allow for the genetic programs that regulate diapause dormancy to be viewed in relative isolation from those that respond to stressors such as anoxia that induce quiescence. Thus, from a mechanistic point of view, these experiments are necessary to clearly outline the discrete responses associated with each state of dormancy and will be highly useful for future studies of embryos responding to natural conditions.

Despite being in a profound state of metabolic depression, with severely reduced rates of protein synthesis [21,47,48], diapause II embryos respond to anoxia with over 500 differentially expressed transcripts, 75% of which increase in abundance. This is a surprising result given the metabolic and energetic status of diapausing embryos and the fact that they do not experience an increase in heat production during exposure to anoxia [26,48]. RNA synthesis can account for 1–15% of the basal metabolic rate of a cell/organism [49,50,51], and thus unnecessary transcription would presumably come at a significant cost to embryos already in an energy limited state. It is possible that this transcriptomic response is at least partially responsible for the 50% drop in ATP levels during the first 14 h of anoxic exposure [26]. Given that RNAseq measures standing stocks of transcripts, these data could be due to de novo synthesis or differential stabilization of transcripts. Stabilization of transcripts has been observed in other anoxia-tolerant systems [52] and thus likely explains at least some of the patterns presented here. However, a robust transcriptional response is a conserved aspect of the cellular stress response [53], and increases in transcripts have been reported in response to anoxia in anoxia-tolerant turtles [54]. The fact that many of the genes upregulated in this study are known to be important in the cellular stress response adds further support for a role of de novo synthesis. Thus, evidence suggests that de novo synthesis of genes is likely; however, an assay specifically measuring new transcripts is needed to be conclusive. How these embryos can mount such a robust response in the face of severe limitations on ATP production is a topic worthy of future exploration.

Recovery from exposure to 24 h of anoxia appears to be an active process with many shared characteristics of anoxic embryos. In early recovery, there are 399 upregulated transcripts and 188 downregulated transcripts; 73% of the upregulated transcripts and 47% of the downregulated transcripts are shared with embryos after 24 h of anoxia. The shared transcripts between early recovery and 24 h of anoxia may indicate the importance of these genes in supporting an adaptive response to reoxygenation, a time when oxidative damage is known to have serious deleterious effects in most organisms [55]. After 24 h of recovery from anoxia, there is only one upregulated transcript, while 47 are downregulated. The upregulated transcript is predicted to be *tsetseEP-like*, a protein with unknown function in vertebrates. Only a single gene ontology term is still overrepresented after 24 h of recovery from anoxia, the downregulation of chaperone binding—perhaps indicating a return to non-stressful conditions, as is also supported by the PCA analysis.

The initial response to acute anoxia (4 h time point) includes a predictable upregulation of transcripts associated with supporting proper protein folding and preventing protein denaturation. The most upregulated transcripts encode for a number of heat shock proteins of the hsp30, 40, 70, and 90 kDa classes and account for five of the top 10 gene ontology terms associated with upregulated genes at 4 h of anoxia. These proteins mediate the folding and compartmentalization of nascent proteins, maintain proteins in functional conformations, protect proteins from irreversible denaturation during stress, aid in their refolding, and assist in the destruction of non-salvageable denatured proteins [4]. After 24 h of anoxia, the initial heat shock response observed at 4 h of anoxia expands to include the endoplasmic reticulum (ER) unfolded protein response (UPR) and the integrated stress response (ISR). The genes associated with the integrated stress response include *MAP3K20*, *DDIT3*, *HSPA5*, *JUN*, *PPP1R15*, *TMEM33*, and *EIF2AK1*. The ISR involves the phosphorylation of *eIF2α*, which causes an immediate decrease in protein synthesis, but interestingly also leads to the increase in translation of the transcription factor ATF4. ATF4 expression is involved with balancing two contradictory outcomes of the ISR, pro-survival and cellular apoptosis [56]. The expression of these upregulated genes is consistent with an activation of the ISR to support survival and recovery [57]. Interestingly, the transcript for *PPP1R15A* increases in abundance after 24 h of anoxia. This protein dephosphorylates eIF2α to regulate translation and modulate the ISR [58], suggesting an initial upregulation of the ISR after 4 h of anoxia and an attenuation of this early response by 24 h of anoxia to perhaps allow for protein synthesis needed to respond to anoxic insult. Finally, the ISR is modified during recovery from anoxia by the additional up-regulation of two key genes, *FOS* and *JUN*. Both *FOS* and *JUN* are members of the AP-1 complex that is activated by p38MAPK in response to oxidative stress [59]. AP-1 proteins may also interact with CHOP (encoded by the *DDIT3* gene) to regulate AP-1 targets in response to stress [60]. When the AP-1 complex interacts with CHOP, cell proliferation and survival are supported through increased expression of BCL-2 and cyclins D and B in human lung tissue exposed to hypoxia [61]. Thus, it appears that AP-1 is likely interacting with the activated ISR to promote return to cell proliferation during recovery from anoxia.

Interestingly, only two protein-coding transcripts, regulator of G-protein signaling 2 (*RGS2*) and taxilin γ (*TXLNG*), were significantly downregulated within the first 4 h of anoxia. RGS2 is a protein that inhibits MAPK3 and AKT signaling in zebrafish [62] and contributes to translational arrest in dormant cancer cells [63]. RGS2 has been shown to inhibit translation through direct interactions with eIF2B as well by interfering with the eIF2–eIF2B GTPase cycle required for initiation of translation [64].Thus, elevated RGS2 in normoxic diapause II embryos may be acting as a brake on protein synthesis to aid in the maintenance of diapause, and this brake may need to be removed for the embryos to respond to anoxic insult. Taxilin γ (TXLNG) can inhibit ATF4 activity and the inflammatory effects of TNFα [65]. *TXLNG* decreased in mouse brain slices in response to hypoxia, which led to activation of ATF4 and the ER unfolded protein response [66]. Thus, we hypothesize that early downregulation of *RGS2* and *TXLNG* in response to anoxia may contribute to the activation of ATF4 and the induction of the ISR as discussed above, making ATF4 a key mediator of the response to anoxia in this species.

p38MAPK appears to be activated within the first few hours of anoxia based on the increase in the relative abundance of several of its key target genes, including *ZFP36L1*, *GADD45A*, and *GADD45G*. By 24 h of anoxia, a total of six genes associated with p38MAPK signaling are upregulated, with *GADD45B*, *DUSP1*, and *MAP3K20* being added to the list. The later upregulation of *DUSP1*, a phosphatase and inhibitor of MAPK [67] that is induced through activation of *P53* in colon cancer cells, may suggest attenuation of the MAPK response after 24 h of anoxia. Conversely, upregulation of *MAP3K20* (*MLK7*), a protein kinase that activates p38MAPK and JNK pathways, would support continued p38MAPK activity if increased transcripts indeed lead to protein expression [68]. The complex nature of MAPK signaling and regulation will require additional experiments to confirm a true role for p38MAPK in supporting anoxia tolerance. It is worth noting that p38MAPK is downregulated in anoxic turtle brains [69], and thus increased activity of p38MAPK in *A. limnaeus* could be a unique adaptation in this species. p38MAPK has been associated with cell cycle regulation and apoptosis pathways through interactions with the transcription factor E2F and anti-apoptotic genes *MCL1* and *BCL2* in other systems [70], and we predict that future studies will elucidate a major role for this protein in the response to anoxia in killifish embryos.

In many tissue types, activation of p38MAPKs during stress pushes cells towards apoptosis, but in some instances, activation promotes cell survival [71,72]. Many of the genes that increased in abundance in response to anoxia are involved with regulation of apoptosis, and many of them are anti-apoptotic (for example MCL-1 [67]). There also appear to be connections between p38MAPK signaling, the ISR, and regulation of apoptosis [73,74]. Embryos of *A. limnaeus* survive short-term exposures to anoxia with little or no apoptosis, suggesting mechanisms that can block apoptosis in response to oxygen stress in comparison to mammals and other oxygen-sensitive species [75,76]. Further, apoptosis is often activated when cells or organisms experience reoxygenation stress [77,78,79,80], but in diapause II embryos, the expression of apoptotic genes remains stable during the initial hours of recovery form anoxia. This suggests that the complement of apoptosis-regulating transcripts is likely appropriate for avoiding apoptosis during recovery. Thus, the pathways involved in regulating apoptosis in these embryos likely reflect a complex balance between pro- and anti-apoptotic signals. The obvious outcome of these interactions is a lack of apoptosis, making diapause II embryos of *A. limnaeus* a potentially powerful model for probing crosstalk and interactions between pro- and anti-apoptotic signaling in response to oxygen limitation.

Major alterations in the transcriptome for lipid homeostasis occur during exposure to anoxia and during initial recovery in diapause II embryos of *A. limnaeus*. Several lipid metabolism ontologies, including glycerolipid metabolic process, cholesterol homeostasis, vesicle organization, and endosomal transport, are overrepresented in upregulated transcripts of embryos during exposure to anoxia and during the initial hours of recovery. The key genes associated with these ontologies are *APOE*, *LIPE*, *SIK1*, and *PCK1*. Salt-inducible kinase 1 (*SIK1*) can inhibit liver lipogenesis in humans [81] and is highly expressed in the ventricle tissue of western painted turtles in anoxia [82]. Lipase e (hormone sensitive) and apolipoprotein E are both involved with the catabolism of lipids, including triacylglycerols, and cholesteryl and retinyl ester bonds [83,84,85]. Furthermore, the cytosolic form of phosphoenolpyruvate carboxykinase 1 (encoded by the gene *PCK1*), is a key enzyme involved in mediating transfer of metabolites and reducing equivalents between the cytosol and mitochondrial compartments to support processes such as gluconeogenesis and glyceroneogenesis [86]. There are also a number of genes involved in vesicular transport and endocytosis [87,88] that are upregulated after 2 h of aerobic recovery. Proper regulation of endosomal membrane transport is known to be regulated during stress by the p38MAPK pathway [89]. Genes that regulate autophagosome dynamics, mitophagy, and the proper handling of ubiquitylated proteins are also upregulated during recovery from anoxia [90,91,92].

There is a notable absence of a transcriptomic signature associated with hypoxia inducible factor (HIF) signaling in diapause II embryos of *A. limnaeus* exposed to anoxia. In fact, HIF signaling pathways are not observed until aerobic recovery from anoxia. We hypothesize that the upregulation of *ZFP36L1* may play a role in blocking HIF signaling in this system. ZFP36L1 is known to target mRNA 3′UTRs and lead to their degradation; *HIF1A* is a known target of *ZFP36L1* [93].

Gene ontology terms associated with transcripts that decrease in abundance during exposure to anoxia and the initial recovery from anoxia are associated with embryonic development, mitochondrial metabolism, and negative regulation of lipase activity. For example, both *SOX8* and *SOX9* are of key importance to embryonic development and are downregulated during anoxia. Many more transcripts that are involved in embryonic development are downregulated during recovery. This list of transcripts includes multiple transcription factors related to development, including *POU3F4*, *SOX7*, *SOX8*, *SOX9*, *SOX18*, *SP8*, and *HOXA9* [94,95,96,97]. The downregulation of these developmental transcription factors may be vital in ensuring normal development while recovering from anoxic stress and ensuring maintenance of diapause. VDR signaling plays a pivotal role in regulation of active development and entrance into diapause in embryos of *A. limnaeus* [15]. The expression of *PIM1* during anoxia with a significant decrease during recovery is an interesting finding given the known interaction between PIM1 and the VDR [98]. *PIM1* is a serine/threonine kinase that supports stem cell proliferation and is protective against ROS induced cell death [99], likely through phosphorylation/inhibition of BAD. The roles of VDR signaling during diapause and in response to anoxia are currently unexplored, but it is intriguing to explore a possible role for *PIM1* and the VDR in the response of diapause embryos to stress.

Transcription factor analysis predicts PPARA as a potential master regulator of the response to anoxia in diapause II embryos of *A. limnaeus*. PPARA is a nuclear hormone receptor known to mediate adaptive responses to fasting and to regulate a variety of metabolic pathways [100,101]. Interestingly, a transcription factor with nine targets in the gene list, CBFA2/RUNX1 partner transcriptional co-repressor 3 (CBFA2T3), is a PPARA-regulated gene that modulates metabolic stress in mouse liver [102]. Thus, between PPARA and CBFA2T3, many of the genes that change significantly during the early response to anoxia (4 h anoxia) can be accounted for, suggesting a large role for PPARA in controlling the immediate response to anoxia. To our knowledge, this is the first implication of PPARA in regulating a response to anoxia.

## 5. Conclusions

This report represents the first transcriptomic analysis of diapausing vertebrate embryos exposed to stress. Despite being in a state of developmental dormancy, diapausing embryos respond to anoxic stress with a robust transcriptomic response that begins shortly after exposure and develops over 24 h. Within hours of anoxic exposure, HSPs and other molecular chaperones are transcriptionally activated, there is a shift expression of genes involved in lipid metabolism, and the p38MAPK signaling pathway is activated. This initial response may be at least partially coordinated by the downregulation of two transcripts, *RGS2* and *TXLNG*, which likely play a significant role in the regulation of translation. This initial response is the foundation of the continued response that develops over the next 20 h. Recovery from anoxia appears to require downregulation of transcripts involved in supporting active development and morphogenesis and may be a response to keep the embryo in diapause. The overarching response to anoxia is an impressive stabilization of 97% of the transcriptome in response to large-scale declines in ATP. In terms of the question of whether diapause II embryos are pre-adapted to survive anoxic stress, the answer appears to be yes, but only partially. While the normoxic transcriptome appears to contain an enrichment of expressed genes that are known to participate in the response to stressful insults, the 3% of transcripts that do change in response to anoxia highlight some interesting and likely essential pathways that must augment any preparations made prior to entrance into dormancy. Perhaps pre-diapause preparation supports stress tolerance in general, and the ability to mount a stress-specific response is retained to tune the response to the particular stress experienced. Future studies may be able to tease these two programs apart by exposing embryos to a variety of stresses and looking for common and unique responses.

## Figures and Tables

**Table 1:** Transcription factors identified by TFLink that are associated with patterns of differential gene expression in response to anoxia and recovery from anoxia in diapause II embryos of *Austrofundulus limnaeus*. Number of associated genes is the number of genes from the gene list that were associated with the transcription factor.

Transcription Factor	Number of Associated Genes	Gene List	*p* Value	*p* Adjust
**4 h anoxia**				
PPARA	5	25	2.13 × 10^−8^	2.00 × 10^-5^
POU4F1	1	25	1.52 × 10^−6^	4.74 × 10^-4^
ZNF746	1	25	1.52 × 10^−6^	4.74 × 10^-4^
HSF2	4	25	7.71 × 10^−6^	1.83 × 10^-3^
POU3F1	1	25	1.51 × 10^−5^	2.84 × 10^-3^
TFAP2B	1	25	2.67 × 10^−5^	3.54 × 10^-3^
DICER1	2	25	4.43 × 10^−5^	7.28 × 10^-3^
**24 h anoxia**				
TFAP4	366	409	4.80 × 10^−14^	1.17 × 10^−11^
TBP	376	409	1.23x 10^−13^	2.50 × 10^−11^
KLF5	347	409	2.18 × 10^−12^	3.82 × 10^−10^
SMAD1	323	409	5.72 × 10^−12^	8.74 × 10^−10^
E2F1	383	409	8.09 × 10^−12^	1.11 × 10^−9^
SNAI2	302	409	1.07 × 10^−11^	1.30 × 10^−9^
SP5	351	409	1.75 × 10^−11^	1.94 × 10^−9^
SMARCA4	401	409	1.98 × 10^−11^	2.01 × 10^−9^
EED	348	409	2.30 × 10^−11^	2.16 × 10^−9^
CEBPA	381	409	3.19 × 10^−11^	2.79 × 10^−9^
**2 h recovery**				
SIN3A	375	405	1.06 × 10^−14^	2.58 × 10^−12^
TAF1	369	405	1.92 × 10^−14^	3.72 × 10^−12^
TBP	374	405	2.15 × 10^−14^	3.72 × 10^−12^
CHD2	339	405	3.54 × 10^−14^	5.35 × 10^−12^
SP4	322	405	3.45 × 10^−13^	4.63 × 10^−11^
KLF9	339	405	4.06 × 10^−13^	4.91 × 10^−11^
SP1	390	405	4.87 × 10^−13^	5.35 × 10^−11^
TFAP4	360	405	7.99 × 10^−13^	8.05 × 10^−11^
MYCN	381	405	9.70 × 10^−13^	9.02 × 10^−11^
E2F1	381	405	1.19 × 10^−12^	1.03 × 10^−10^
**24 h recovery**				
XRCC6	1	42	4.35 × 10^−6^	4.24 × 10^−3^
TRAF6	1	42	1.30 × 10^−5^	6.36 × 10^−3^

## Data Availability

The sequencing data files, in FASTQ format, are available on the NCBI’s website. https://www.ncbi.nlm.nih.gov/Traces/study/?acc=PRJNA272154&o=acc_s%3Aa (accessed on 31 March 2025), is the web address to directly access our lab’s uploaded sequencing files. At the time of submission, metadata for the samples presented in this paper were not assigned on the NCBI website, but these metadata and accession numbers are contained in Table S1. Metadata for samples presented in the paper will be updated on the NCBI website in a timely manner.

## Supplementary Materials

The following supporting information can be downloaded at https://www.mdpi.com/article/10.3390/biom15040515/s1: Table S1: cDNA library results; Table S2: Highly expressed T0 transcripts; Table S3: Gene ontology terms enriched in highly expressed genes in diapause II embryos of *Austrofundulus limnaeus*; Table S4: Differential gene expression patterns for diapause II embryos of *Austrofundulus limnaeus* exposed to anoxia and recovery from anoxia; Table S5: qPCR validation of RNAseq differential expression analysis; Table S6: KEGG pathway analysis for genes that increase in abundance in response to anoxia and recovery from anoxia in diapause II embryos of *Austrofundulus limnaeus*; Table S7: Gene ontology enrichment analysis for genes that increase in abundance in response to anoxia and recovery from anoxia in diapause II embryos in *Austrofundulus limnaeus*; Table S8: Gene ontology enrichment analysis for genes that decrease in abundance in response to anoxia and recovery from anoxia in diapause II embryos of *Austrofundulus limnaeus*; Table S9: KEGG pathway analysis for genes that decrease in abundance in response to anoxia and recovery from anoxia in diapause II embryos of *Austrofundulus limnaeus*; Table S10: Transcription factor analysis of differentially expressed genes.

## Abbreviations

The following abbreviations are used in this manuscript:

dpf	days post fertilization
NCBI	The National Center for Biotechnology Information
ORA	overrepresentation analysis
TFs	transcription factors
ER	endoplasmic reticulum
UPR	unfolded protein response
ISR	integrated stress response

## References

[1] Storey K.B., Storey J.M. (1990). Metabolic rate depression and biochemical adaptation in anaerobiosis, hibernation and estivation. Q. Rev. Biol..

[2] Storey K.B. (1988). Suspended animation: The molecular basis of metabolic depression. Can. J. Zool..

[3] Hand S.C., Gilles R. (1991). Metabolic dormancy in aquatic invertebrates. Advances in Comparative and Environmental Physiology.

[4] MacRae T.H. (2010). Gene expression, metabolic regulation and stress tolerance during diapause. Cell. Mol. Life Sci..

[5] Denlinger D.L. (2023). Insect diapause: From a rich history to an exciting future. J. Exp. Biol..

[6] Sim C., Kang D.S., Kim S., Bai X., Denlinger D.L. (2015). Identification of FOXO targets that generate diverse features of the diapause phenotype in the mosquito *Culex pipiens*. Proc. Natl. Acad. Sci. USA.

[7] Rinehart J.P., Yocum G.D., Denlinger D.L. (2000). Developmental upregulation of inducible hsp70 transcripts, but not the cognate form, during pupal diapause in the flesh fly, *Sarcophaga crassipalpis*. Insect Biochem. Mol. Biol..

[8] Rinehart J.P., Denlinger D.L. (2000). Heat-shock protein 90 is down-regulated during pupal diapause in the flesh fly, *Sarcophaga crassipalpis*, but remains responsive to thermal stress. Insect Mol. Biol..

[9] Tammariello S.P., Rinehart J.P., Denlinger D.L. (1999). Desiccation elicits heat shock protein transcription in the flesh fly, *Sarcophaga crassipalpis*, but does not enhance tolerance to high or low temperatures. J. Insect Physiol..

[10] Yocum G., Joplin K., Denlinger D. (1998). Upregulation of a 23 kDa small heat shock protein transcript during pupal diapause in the flesh fly, *Sarcophaga crassipalpis*. Insect Biochem. Mol. Biol..

[11] Podrabsky J., Riggs C., Wagner J., Berois N., García G., De Sá R. (2016). Tolerance of Environmental Stress. Annual Fishes. Life History Strategy, Diversity, and Evolution.

[12] Podrabsky J., Romney A., Culpepper K., Berois N., García G., De Sá R. (2016). Alternative Developmental Pathways. Annual Fishes. Life History Strategy, Diversity, and Evolution.

[13] Wourms J.P. (1972). The developmental biology of annual fishes III. Pre-embryonic and embryonic diapause of variable duration in the eggs of annual fishes. J. Exp. Zool..

[14] Podrabsky J., Riggs C., Romney A., Woll S., Wagner J., Culpepper K., Cleaver T. (2017). Embryonic development of the annual killifish *Austrofundulus limnaeus*: An emerging model for ecological and evolutionary developmental biology research and instruction. Dev. Dyn..

[15] Romney A., Davis E., Corona M., Wagner J., Podrabsky J. (2018). Temperature dependent vitamin D signaling regulates developmental trajectory associated with diapause in an annual killifish. Proc. Natl. Acad. Sci. USA.

[16] Podrabsky J., Wilson N. (2016). Hypoxia and anoxia tolerance in the annual killifish *Austrofundulus limnaeus*. Integr. Comp. Biol..

[17] Podrabsky J.E., Lopez J.P., Fan T.W.M., Higashi R., Somero G.N. (2007). Extreme anoxia tolerance in embryos of the annual killifish *Austrofundulus limnaeus*: Insights from a metabolomics analysis. J. Exp. Biol..

[18] Machado B.E., Podrabsky J.E. (2007). Salinity tolerance in diapausing embryos of the annual killifish *Austrofundulus limnaeus* is supported by exceptionally low water and ion permeability. J. Comp. Physiol. B.

[19] Podrabsky J.E., Riggs C.L., Duerr J.M., Padilla P. (2012). Anoxia Tolerance During Vertebrate Development—Insights from Studies on the Annual Killifish *Austrofundulus limnaeus*. Anoxia.

[20] Hand S.C., Podrabsky J.E. (2000). Bioenergetics of diapause and quiescence in aquatic animals. Thermochim. Acta.

[21] Podrabsky J.E., Hand S.C. (2000). Depression of protein synthesis during diapause in embryos of the annual killifish *Austrofundulus limnaeus*. Physiol. Biochem. Zool..

[22] Polačik M., García D., Arezo M.J., Papa N., Schlueb H., Blanco D., Podrabsky J.E., Vrtílek M. (2023). Embryonic development of natural annual killifish populations of the genus *Austrolebias*: Evolutionary parallelism and the role of environment. Freshw. Biol..

[23] Polačik M., Vrtílek M., Reichard M., Žák J., Blažek R., Podrabsky J. (2021). Embryo ecology: Developmental synchrony and asynchrony in the embryonic development of wild annual fish populations. Ecol. Evol..

[24] Zajic D., Nicholson J., Podrabsky J. (2020). No water, no problem: Stage-specific metabolic responses to dehydration stress in annual killifish embryos. J. Exp. Biol..

[25] Riggs C., Podrabsky J. (2017). Small noncoding RNA expression during extreme anoxia tolerance of annual killifish (*Austrofundulus limnaeus*) embryos. Physiol. Genom..

[26] Podrabsky J.E., Menze M.A., Hand S.C. (2012). Rapid Communication: Long-term survival of anoxia despite rapid ATP decline in embryos of the annual killifish *Austrofundulus limnaeus*. J. Exp. Zool. A Ecol. Genet. Physiol..

[27] Podrabsky J.E. (1999). Husbandry of the annual killifish *Austrofundulus limnaeus* with special emphasis on the collection and rearing of embryos. Env. Biol. Fishes.

[28] Wourms J.P. (1972). The developmental biology of annual fishes I. Stages in the normal development of *Austrofundulus myersi* Dahl. J. Exp. Zool..

[29] Meller C.L., Meller R., Simons R.P., Podrabsky J.E. (2014). Patterns of ubiquitylation and SUMOylation associated with exposure to anoxia in embryos of the annual killifish *Austrofundulus limnaeus*. J. Comp. Physiol. B.

[30] Chomczynski P., Sacchi N. (1987). Single-step method of RNA isolation by acid guanidinium thiocyanate-phenol-chloroform extraction. Anal. Biochem..

[31] Chomczynski P., Sacchi N. (2006). The single-step method of RNA isolation by acid guanidinium thiocyanate–phenol–chloroform extraction: Twenty-something years on. Nat. Protoc..

[32] Chomczynski P., Mackey K. (1995). Short technical reports. Modification of the TRI reagent procedure for isolation of RNA from polysaccharide-and proteoglycan-rich sources. Biotechniques.

[33] Wagner J.T., Podrabsky J.E. (2015). Gene expression patterns that support novel developmental stress buffering in embryos of the annual killifish *Austrofundulus limnaeus*. EvoDevo.

[34] Romney A., Podrabsky J. (2017). Transcriptomic analysis of maternally provisioned cues for phenotypic plasticity in the annual killifish, *Austrofundulus limnaeus*. EvoDevo.

[35] Andrews S. FastQC: A Quality Control Tool for High Throughput Sequence Data. https://www.bioinformatics.babraham.ac.uk/projects/fastqc/.

[36] Bolger A.A., Lohse M., Usadel B. (2014). Trimmomatic: A flexible trimmer for Illumina Sequence Data. Bioinformatics.

[37] Langmead B., Salzberg S.L. (2012). Fast gapped-read alignment with Bowtie 2. Nat. Methods.

[38] Gentleman R.C., Carey V.J., Bates D.M., Bolstad B., Dettling M., Dudoit S., Ellis B., Gautier L., Ge Y., Gentry J. (2004). Bioconductor: Open software development for computational biology and bioinformatics. Genome Biol..

[39] Wagner J., Warren W., Minx P., Podrabsky J. Austrofundulus limnaeus 1.0 Draft Genome Assembly with Annotation. http://www.ncbi.nlm.nih.gov/genome/?term=txid52670[orgn].

[40] Love M.I., Huber W., Anders S. (2014). Moderated estimation of fold change and dispersion for RNA-seq data with DESeq2. Genome Biol..

[41] Emms D.M., Kelly S. (2019). OrthoFinder: Phylogenetic orthology inference for comparative genomics. Genome Biol..

[42] Wu T., Hu E., Xu S., Chen M., Guo P., Dai Z., Feng T., Zhou L., Tang W., Zhan L. (2021). clusterProfiler 4.0: A universal enrichment tool for interpreting omics data. Innovation.

[43] Liska O., Bohár B., Hidas A., Korcsmáros T., Papp B., Fazekas D., Ari E. (2022). TFLink: An integrated gateway to access transcription factor–target gene interactions for multiple species. Database.

[44] Podrabsky J.E., Somero G.N. (2007). An inducible 70 kDa-class heat shock protein is constitutively expressed during early development and diapause in the annual killifish *Austrofundulus limnaeus*. Cell Stress Chaperones.

[45] Stricher F., Macri C., Ruff M., Muller S. (2013). HSPA8/HSC70 chaperone protein. Autophagy.

[46] Wang C., Zhang X., Wang X., Zhai Y., Li M., Pan J., Bai Y., Rong X., Zhou J. (2022). Genetic deletion of *hspa8* leads to selective tissue malformations in zebrafish embryonic development. J. Cell Sci..

[47] Podrabsky J.E., Hand S.C. (2015). Physiological strategies during animal diapause: Lessons from brine shrimp and annual killifish. J. Exp. Biol..

[48] Podrabsky J.E., Hand S.C. (1999). The bioenergetics of embryonic diapause in an annual killifish, *Austrofundulus limnaeus*. J. Exp. Biol..

[49] Buttgereit F., Brand M.D. (1995). A hierarchy of ATP-consuming processes in mammalian cells. Biochem. J..

[50] Buttgereit F., Brand M.D., Muller M. (1992). ConA induced changes in energy metabolism of rat thymocytes. Biosci. Rep..

[51] Rolfe D.F.S., Brown G.C. (1997). Cellular energy utilization and molecular origin of standard metabolic rate in mammals. Physiol. Rev..

[52] Hardewig I., Anchordoguy T.J., Crawford D.L., Hand S.C. (1996). Profiles of nuclear and mitochondrial encoded mRNAs in developing and quiescent embryos of *Artemia franciscana*. Mol. Cell Biochem..

[53] Morimoto R.I. (1998). Regulation of the heat shock transcriptional response: Cross talk between a family of heat shock factors, molecular chaperones, and negative regulators. Genes Dev..

[54] Milton S.L., Nayak G., Lutz P.L., Prentice H.M. (2006). Gene transcription of neuroglobin is upregulated by hypoxia and anoxia in the brain of the anoxia-tolerant turtle Trachemys scripta. J. Biomed. Sci..

[55] Bundgaard A., Ruhr I.M., Fago A., Galli G.L. (2020). Metabolic adaptations to anoxia and reoxygenation: New lessons from freshwater turtles and Crucian carp. Curr. Opin. Endocr. Metab. Res..

[56] Neill G., Masson G.R. (2023). A stay of execution: ATF4 regulation and potential outcomes for the integrated stress response. Front. Mol. Neurosci..

[57] Suragani R.N.V.S., Zachariah R.S., Velazquez J.G., Liu S., Sun C.-W., Townes T.M., Chen J.-J. (2012). Heme-regulated eIF2α kinase activated Atf4 signaling pathway in oxidative stress and erythropoiesis. Blood.

[58] Crespillo-Casado A., Chambers J.E., Fischer P.M., Marciniak S.J., Ron D. (2017). PPP1R15A-mediated dephosphorylation of eIF2α is unaffected by Sephin1 or Guanabenz. eLife.

[59] Dharshini P.L.C., Vishnupriya S., Sakthivel K.M., Rasmi R.R. (2020). Oxidative stress responsive transcription factors in cellular signalling transduction mechanisms. Cell Signal.

[60] Ubeda M., Vallejo M., Habener J.F. (1999). CHOP enhancement of gene transcription by interactions with Jun/Fos AP-1 complex proteins. Mol. Cell Biol..

[61] Yadav S., Kalra N., Ganju L., Singh M. (2017). Activator protein-1 (AP-1): A bridge between life and death in lung epithelial (A549) cells under hypoxia. Mol. Cell Biochem..

[62] Lin S.-J., Huang Y.-C., Chen H.-Y., Fang J.-Y., Hsu S.-Y., Shih H.-Y., Liu Y.-C., Cheng Y.-C. (2021). RGS2 suppresses melanoma growth via inhibiting MAPK and AKT signaling pathways. Anticancer. Res..

[63] Cho J., Min H.-Y., Lee H.J., Hyun S.Y., Sim J.Y., Noh M., Hwang S.J., Park S.-H., Boo H.-J., Lee H.-J. (2021). RGS2-mediated translational control mediates cancer cell dormancy and tumor relapse. J. Clin. Investig..

[64] Nguyen C.H., Ming H., Zhao P., Hugendubler L., Gros R., Kimball S.R., Chidiac P. (2009). Translational control by RGS2. J. Cell Biol..

[65] Tao J., Gu P., Lai H., Peng H., Guo Z., Yuan Y., Yu X., Shen X., Liu J., Xier Z. (2023). TXLNG improves insulin resistance in obese subjects in vitro and in vivo by inhibiting ATF4 transcriptional activity. Mol. Cell Endocrinol..

[66] Hotokezaka Y., Katayama I., Nakamura T. (2020). ATM-associated signalling triggers the unfolded protein response and cell death in response to stress. Commun. Biol..

[67] Liu Y.-X., Wang J., Guo J., Wu J., Lieberman H.B., Yin Y. (2008). *DUSP1* is controlled by p53 during the cellular response to oxidative stress. Mol. Cancer Res..

[68] Wang X., Mader M.M., Toth J.E., Yu X., Jin N., Campbell R.M., Smallwood J.K., Christe M.E., Chatterjee A., Goodson T. (2005). Complete inhibition of anisomycin and UV radiation but not cytokine induced JNK and p38 activation by an aryl-substituted dihydropyrrolopyrazole quinoline and mixed lineage kinase 7 small interfering RNA. J. Biol. Chem..

[69] Milton S.L., J Dirk L., F Kara L., Prentice H.M. (2008). Adenosine modulates ERK1/2, PI3K/Akt, and p38MAPK activation in the brain of the anoxia-tolerant turtle Trachemys scripta. J. Cereb. Blood Flow. Metab..

[70] Whitaker R.H., Cook J.G. (2021). Stress relief techniques: p38 MAPK determines the balance of cell cycle and apoptosis pathways. Biomolecules.

[71] Cargnello M., Roux P.P. (2011). Activation and function of the MAPKs and their substrates, the MAPK-activated protein kinases. Microbiol. Mol. Biol..

[72] Gupta M., Gupta S.K., Hoffman B., Liebermann D.A. (2006). Gadd45a and Gadd45b protect hematopoietic cells from UV-induced apoptosis via distinct signaling pathways, including p38 activation and JNK inhibition. J. Biol. Chem..

[73] Joshi S. (2014). Mnk kinase pathway: Cellular functions and biological outcomes. World J. Biol. Chem..

[74] Sheikh S., Saxena D., Tian X., Amirshaghaghi A., Tsourkas A., Brem S., Dorsey J.F. (2019). An integrated stress response agent that modulates DR5-dependent TRAIL synergy reduces patient-derived glioma stem cell viability. Mol. Cancer Res..

[75] Culpepper K.M., Podrabsky J.E. (2012). Cell cycle regulation during development and dormancy in embryos of the annual killifish *Austrofundulus limnaeus*. Cell Cycle.

[76] Meller C.L., Podrabsky J.E. (2013). Avoidance of apoptosis in embryonic cells of the annual killifish *Austrofundulus limnaeus* exposed to anoxia. PLoS ONE.

[77] Lesnefsky E.J., Chen Q., Tandler B., Hoppel C.L. (2017). Mitochondrial dysfunction and myocardial ischemia-reperfusion: Implications for novel therapies. Annu. Rev. Pharmacol. Toxicol..

[78] Hochrainer K., Jackman K., Anrather J., Iadecola C. (2012). Reperfusion rather than ischemia drives the formation of ubiquitin aggregates after middle cerebral artery occlusion. Stroke.

[79] Braunersreuther V., Jaquet V. (2012). Reactive oxygen species in myocardial reperfusion injury: From physiopathology to therapeutic approaches. Curr. Pharm. Biotechnol..

[80] Kalogeris T., Baines C.P., Krenz M., Korthuis R.J. (2012). Cell biology of ischemia/reperfusion injury. Int. Rev. Cell Mol. Biol..

[81] Zhang Y., Takemori H., Wang C., Fu J., Xu M., Xiong L., Li N., Wen X. (2017). Role of salt inducible kinase 1 in high glucose-induced lipid accumulation in HepG2 cells and metformin intervention. Life Sci..

[82] Keenan S.W., Hill C.A., Kandoth C., Buck L.T., Warren D.E. (2015). Transcriptomic responses of the heart and brain to anoxia in the Western Painted Turtle. PLoS ONE.

[83] Grober J., Lucas S., Sörhede-Winzell M., Zaghini I., Mairal A., Contreras J.-A., Besnard P., Holm C., Langin D. (2003). Hormone-sensitive lipase is a cholesterol esterase of the intestinal mucosa. J. Biol. Chem..

[84] Huang Y., Mahley R.W. (2014). Apolipoprotein E: Structure and function in lipid metabolism, neurobiology, and Alzheimer’s diseases. Neurobiol. Dis..

[85] Khalil Y.A., Rabès J.-P., Boileau C., Varret M. (2021). APOE gene variants in primary dyslipidemia. Atherosclerosis.

[86] Nye C.K., Hanson R.W., Kalhan S.C. (2008). Glyceroneogenesis is the dominant pathway for triglyceride glycerol synthesis in vivo in the rat. J. Biol. Chem..

[87] Chatterjee S., Choi A.J., Frankel G. (2021). A systematic review of Sec24 cargo interactome. Traffic.

[88] Linders P.T., Van Der Horst C., Ter Beest M., Van Den Bogaart G. (2019). Stx5-mediated ER-Golgi transport in mammals and yeast. Cells.

[89] López-Hernández T., Haucke V., Maritzen T. (2020). Endocytosis in the adaptation to cellular stress. Cell Stress.

[90] Park J., Kim J., Park H., Kim T., Lee S. (2024). ESCRT-III: A versatile membrane remodeling machinery and its implications in cellular processes and diseases. Anim. Cells Syst..

[91] Kumar A.V., Mills J., Lapierre L.R. (2022). Selective autophagy receptor p62/SQSTM1, a pivotal player in stress and aging. Front. Cell Dev. Biol..

[92] Hu X., Wang L., Wang Y., Ji J., Li J., Wang Z., Li C., Zhang Y., Zhang Z.-R. (2020). RNF126-mediated reubiquitination is required for proteasomal degradation of p97-extracted membrane proteins. Mol. Cell.

[93] Loh X.-Y., Sun Q.-Y., Ding L.-W., Mayakonda A., Venkatachalam N., Yeo M.-S., Silva T.C., Xiao J.-F., Doan N.B., Said J.W. (2020). RNA-binding protein *ZFP36L1* suppresses hypoxia and cell-cycle signaling. Cancer Res..

[94] She Z.-Y., Yang W.-X. (2015). SOX family transcription factors involved in diverse cellular events during development. Eur. J. Cell Biol..

[95] Svingen T., Tonissen K.F. (2006). Hox transcription factors and their elusive mammalian gene targets. Heredity.

[96] Robert-Moreno À., Naranjo S., De La Calle-Mustienes E., Gómez-Skarmeta J.L., Alsina B. (2010). Characterization of new otic enhancers of the Pou3f4 gene reveal distinct signaling pathway regulation and spatio-temporal patterns. PLoS ONE.

[97] Bell S.M., Schreiner C.M., Waclaw R.R., Campbell K., Potter S.S., Scott W.J. (2003). *Sp8* is crucial for limb outgrowth and neuropore closure. Proc. Natl. Acad. Sci. USA.

[98] Maier C.J., Maier R.H., Rid R., Trost A., Hundsberger H., Eger A., Hintner H., Bauer J.W., Onder K. (2012). PIM-1 kinase interacts with the DNA binding domain of the vitamin D receptor: A further kinase implicated in 1,25-(OH)2D3 signaling. BMC Mol. Biol..

[99] Gu J.J., Wang Z., Reeves R., Magnuson N.S. (2009). PIM1 phosphorylates and negatively regulates ASK1-mediated apoptosis. Oncogene.

[100] König B., Rauer C., Rosenbaum S., Brandsch C., Eder K., Stangl G.I. (2009). Fasting upregulates PPAR*α* target genes in brain and influences pituitary hormone expression in a PPAR*α* dependent manner. PPAR Res..

[101] Mandard S., Müller M., Kersten S. (2004). Peroxisome proliferator-activated receptor a target genes. Cell. Mol. Life Sci..

[102] Kim D., Ha S.K., Gonzalez F.J. (2024). CBFA2T3 is PPARA sensitive and attenuates fasting-induced lipid accumulation in mouse liver. Cells.

